# Outcome of Targeted Therapy for Obstructive Sleep Apnea in Children with Obesity and Metabolic Dysfunction-Associated Steatotic Liver Disease (MASLD)

**DOI:** 10.3390/children13091241

**Published:** 2026-09-14

**Authors:** Woranart Ratanakorn

**Affiliations:** Department of Pediatrics, Chonburi Hospital, Chonburi 20000, Thailand; woranartr@gmail.com

**Keywords:** obstructive sleep apnea, metabolic dysfunction-associated steatotic liver disease, adenotonsillectomy, continuous positive airway pressure

## Abstract

**Highlights:**

**What are the main findings?**

- Targeted treatment of pediatric obstructive sleep apnea was associated with favorable changes in liver enzymes, ultrasonographic hepatic steatosis, polysomnographic parameters, and nocturnal oxygenation in children with obesity and MASLD.
- Continuous positive airway pressure (CPAP) therapy was associated with both a ≥30% reduction in ALT and an improvement in ultrasonographic hepatic steatosis, whereas adenotonsillectomy and BMI z-score reduction ≥ 0.25 were not significantly associated with either hepatic outcome.

**What are the implications of the main findings?**

- Assessment and appropriate management of OSA may be an important component of multidisciplinary care for children with obesity and MASLD, particularly for those with persistent liver injury.
- The favorable hepatic outcomes associated with CPAP highlight the potential value of integrating sleep evaluation and OSA management into multidisciplinary care for children with obesity and MASLD.

**Abstract:**

**Background:** Obstructive sleep apnea (OSA) and metabolic dysfunction-associated steatotic liver disease (MASLD) frequently coexist in children with obesity, but data on hepatic outcomes following OSA treatment are limited. This study evaluated associations between targeted OSA treatment and longitudinal changes in sleep-disordered breathing and hepatic outcomes. **Methods:** This retrospective observational cohort included children aged 4–18 years with obesity, polysomnography-confirmed OSA, and ultrasonography-confirmed MASLD who received clinical care between 2018 and 2025. Children received targeted OSA treatment (adenotonsillectomy and/or continuous positive airway pressure [CPAP]) or non-targeted treatment. Polysomnographic parameters, liver enzymes, and ultrasonographic hepatic steatosis were assessed at baseline and 6–12 months. The primary outcome was a ≥30% reduction in alanine aminotransferase (ALT). **Results:** Among 127 children, 67 received targeted treatment and 60 did not. Targeted treatment was associated with improvements in OSA severity, nocturnal oxygenation, ALT, AST, and ultrasonographic steatosis, whereas less favorable trajectories occurred without targeted treatment. ALT reduction differed significantly among treatment modalities, with the greatest reduction observed with CPAP. In multivariable analyses, CPAP was associated with both ≥30% ALT reduction (OR 14.54, 95% CI 3.87–54.69; *p* < 0.01) and ultrasonographic improvement (OR 3.33, 95% CI 1.16–9.59; *p* = 0.03). A BMI z-score reduction of ≥0.25 was not independently associated with either hepatic outcome. **Conclusions:** Targeted OSA treatment was associated with more favorable sleep and hepatic outcomes, with the strongest associations observed for CPAP. Prospective studies are needed to determine whether these associations represent causal treatment effects.

## 1. Introduction

Metabolic dysfunction-associated steatotic liver disease (MASLD), formerly termed nonalcoholic fatty liver disease (NAFLD), is now recognized as the most common chronic liver disease in children and adolescents and an increasingly important complication of pediatric obesity. The 2023 multisociety Delphi process introduced steatotic liver disease as an overarching term and MASLD as the preferred term for steatosis associated with cardiometabolic risk, reflecting the central role of metabolic dysfunction while reducing stigmatizing terminology [1]. Recent European and Asian-Pacific guidelines further support this nomenclature and emphasize risk stratification, cardiometabolic assessment, and longitudinal management [2,3]. Pediatric experts have also highlighted that childhood steatotic liver disease has unique clinical and histological features that require pediatric-specific interpretation rather than direct extrapolation from adult disease [4].

The burden of pediatric MASLD has increased in parallel with childhood obesity. Population-based estimates suggest that fatty liver affects approximately 7–10% of the general pediatric population, with substantially higher prevalence among children with obesity [5,6]. In affected children, disease severity ranges from simple steatosis to metabolic dysfunction-associated steatohepatitis, fibrosis, cirrhosis, and end-stage liver disease [2,7]. Pediatric MASLD is clinically important because liver injury beginning in childhood may persist into adulthood and is associated with insulin resistance, dyslipidemia, type 2 diabetes risk, and long-term cardiometabolic morbidity [8,9,10,11]. The SAFETY study also demonstrated that conventional alanine aminotransferase (ALT) cutoffs may be too high for reliable identification of pediatric chronic liver disease, underscoring the need for early recognition and careful monitoring [12].

The pathogenesis of MASLD is complex and multifactorial. The historical two-hit hypothesis has been replaced by a multiple-hit or multiple-parallel-hit model in which insulin resistance, adipose tissue dysfunction, oxidative stress, mitochondrial injury, inflammatory signaling, genetic susceptibility, gut microbiota alterations, and environmental factors interact to promote hepatic steatosis and progressive liver injury [13,14,15,16,17,18]. Within this framework, sleep-related breathing disorders have gained increasing attention as potentially modifiable contributors to metabolic dysfunction and liver disease progression [19,20,21,22,23,24].

Obstructive sleep apnea (OSA) is characterized by recurrent partial or complete upper-airway obstruction during sleep, producing intermittent hypoxemia, sleep fragmentation, hypercapnia, and negative intrathoracic pressure swings [25,26,27,28]. Although pediatric OSA affects a minority of otherwise healthy children, its prevalence is much higher among children with obesity, with some cohorts reporting OSA in more than half of obese children [29,30,31,32]. Obesity and OSA share overlapping mechanisms, including systemic inflammation, oxidative stress, sympathetic activation, endothelial dysfunction, and insulin resistance, all of which may amplify pathways involved in MASLD progression [19,20,28,29,30,31,32].

A strong mechanistic rationale supports a link between OSA and MASLD. Chronic intermittent hypoxia, a defining physiologic feature of OSA, activates hypoxia-inducible pathways, increases reactive oxygen species production, promotes lipid peroxidation, impairs mitochondrial function, and stimulates inflammatory cytokine release [19,20,33,34,35]. Intermittent hypoxia may also worsen insulin resistance and alter hepatic lipid metabolism, thereby accelerating the transition from steatosis to steatohepatitis and fibrosis [21,22,23,24,33,34,35,36,37,38]. Animal and translational studies consistently demonstrate that intermittent hypoxia can induce hepatic steatosis and inflammatory liver injury, supporting a causal contribution of hypoxic burden to fatty liver disease progression [33,34,35].

Clinical evidence is consistent with these experimental observations. Adult studies and meta-analyses have linked OSA severity and nocturnal hypoxemia with elevated aminotransferase levels, hepatic steatosis, steatohepatitis, and fibrosis [21,22,23,24,36,37,38,39,40,41,42,43]. Pediatric data, although more limited, are particularly relevant. Sundaram and colleagues reported that OSA and hypoxemia were associated with advanced liver histology in children with NAFLD and later showed that intermittent nocturnal hypoxia was associated with pediatric steatohepatitis [44,45]. Nobili et al. similarly demonstrated that pediatric OSA was associated with worse liver histology and inflammatory activation independent of obesity and insulin resistance [46]. Additional pediatric studies and meta-analytic data support associations between apnea–hypopnea index (AHI), oxygen desaturation burden, hepatic steatosis, and liver injury markers among children with obesity [47,48].

Treatment of pediatric OSA is individualized and may include adenotonsillectomy, positive airway pressure therapy, weight management, and adjunctive medical therapy [25,26,28,49,50,51,52]. Adenotonsillectomy improves polysomnographic indices and symptoms in many children, although residual OSA is common in those with obesity [49,50,51,52]. Continuous positive airway pressure (CPAP) is an important treatment for persistent OSA, severe obesity-associated OSA, or for children who are not surgical candidates, but adherence remains a key determinant of effectiveness [53,54]. Beyond respiratory outcomes, OSA treatment may improve cardiometabolic physiology by reducing intermittent hypoxia, sleep fragmentation, sympathetic activation, and inflammatory stress [39,40,41,42,43].

However, whether targeted OSA therapy improves MASLD outcomes in children remains insufficiently defined. Adult CPAP studies have produced heterogeneous results for liver enzymes, steatosis, and fibrosis, ranging from improvement in some cohorts to no significant benefit in randomized data [39,40,41,42,43]. Pediatric longitudinal evidence is scarce, although one study suggested that CPAP treatment of OSA and nocturnal hypoxia may improve severity markers of pediatric NAFLD [55]. Given the high co-occurrence of obesity, OSA, and MASLD, identification of sleep-disordered breathing as a modifiable contributor to liver injury could expand the therapeutic approach beyond lifestyle intervention alone [56,57].

Therefore, this retrospective observational cohort study aimed to evaluate the association between targeted OSA treatment, including adenotonsillectomy and CPAP therapy, and longitudinal changes in polysomnographic parameters, liver enzymes, and hepatic steatosis among children with obesity and MASLD. We also examined associations between OSA severity, nocturnal oxygenation, and markers of liver injury and explored factors associated with biochemical hepatic response during follow-up. We hypothesized that receipt of targeted OSA treatment would be associated with improvements in sleep-disordered breathing, nocturnal oxygenation, and biochemical markers of hepatic injury.

## 2. Materials and Methods

### 2.1. Study Design and Setting

This was a retrospective, observational, real-world cohort study conducted at a single tertiary-care institution: the Pediatric Sleep Clinic of Chonburi Hospital, Thailand. Clinical data from patients treated between 2018 and 2025 were retrospectively obtained from routine medical records. OSA treatment had been determined as part of routine clinical care according to clinical characteristics, polysomnographic findings, physician judgment, and family preference. The study aimed to evaluate the effect of targeted treatment for obstructive sleep apnea (OSA) on hepatic steatosis and liver injury markers in children with obesity and metabolic-associated steatotic liver disease (MASLD). Baseline polysomnographic and liver assessments were performed at the initial evaluation, with repeat assessments conducted after 6–12 months of follow-up.

### 2.2. Study Population

Children aged 4–18 years who were referred to the pediatric sleep clinic for evaluation of suspected sleep-disordered breathing were screened for eligibility. Participants were included if they met the following criteria: (1) obesity, defined as a body mass index (BMI) z-score greater than 2 according to the World Health Organization growth reference; (2) diagnosis of OSA confirmed by overnight polysomnography with an apnea–hypopnea index (AHI) ≥ 1 event per hour of sleep; and (3) evidence of hepatic steatosis on abdominal ultrasonography consistent with MASLD in the presence of obesity. Children were excluded if they had viral hepatitis (hepatitis B or C infection), autoimmune liver disease, inherited metabolic liver disorders, use of hepatotoxic medications, or chronic systemic diseases known to affect liver function.

### 2.3. Targeted Treatment of Obstructive Sleep Apnea

Participants were categorized into two groups according to clinical management of OSA. The targeted treatment group received OSA-specific therapy based on clinical evaluation and polysomnographic findings. Adenotonsillectomy was performed in children with adenotonsillar hypertrophy and moderate-to-severe OSA. Continuous positive airway pressure (CPAP) therapy was prescribed for children with severe OSA who were not surgical candidates, had persistent OSA following adenotonsillectomy, or had contraindications to surgery. CPAP was individually titrated during attended overnight polysomnography to achieve optimal control of obstructive respiratory events. Treatment adherence was objectively assessed using data downloaded from the CPAP device at follow-up visits. Adherence was defined as the percentage of nights during which CPAP was used for at least 4 h. Adequate adherence was defined as CPAP use for ≥4 h per night on ≥70% of monitored nights, consistent with commonly accepted clinical criteria [53]. In addition, average nightly CPAP use (hours/night) was recorded throughout the follow-up period.

Children who did not undergo adenotonsillectomy or CPAP therapy due to parental refusal, medical contraindications, or inability to tolerate CPAP during the initial trial were classified into the non-targeted treatment group. None of the children in this group received CPAP during the follow-up period. Both groups received standard lifestyle interventions, including dietary counseling, weight management recommendations, sleep hygiene education, and encouragement of regular physical activity.

### 2.4. Polysomnography

All participants underwent overnight polysomnography in a dedicated pediatric sleep laboratory using standard monitoring techniques. Recorded signals included electroencephalography, electrooculography, electromyography, electrocardiography, nasal airflow (via pressure transducer and thermistor), thoracoabdominal respiratory effort belts, pulse oximetry, and body position monitoring. Sleep stages and respiratory events were scored according to the criteria established by the American Academy of Sleep Medicine version 3.0, 2023 [27] by a certified pediatric sleep disorders specialist. The apnea–hypopnea index (AHI) was calculated as the number of apneas and hypopneas per hour of sleep. OSA severity was classified as no OSA (AHI < 1 event/h), mild OSA (AHI 1–4.9 events/h), moderate OSA (AHI 5–9.9 events/h), and severe OSA (AHI ≥ 10 events/h) according to pediatric scoring guidelines. Additional polysomnographic parameters included mean oxygen saturation during sleep and nadir oxygen saturation.

### 2.5. Assessment of Hepatic Steatosis

Hepatic steatosis was assessed using abdominal ultrasonography performed by two experienced radiologists who were blinded to treatment status. Steatosis was graded using a standardized semiquantitative scoring system based on liver echogenicity, hepatorenal contrast, and vascular blurring. The severity of steatosis was categorized as grade 0—normal liver echogenicity; grade 1—mild, slightly increased echogenicity (liver echogenicity slightly greater than kidney; vessels and diaphragm remain clearly visible); grade 2—moderate, clearly increased echogenicity with some attenuation (visualization of vessels/diaphragm becomes reduced); grade 3—severe, markedly increased echogenicity with substantial posterior attenuation (vessels and diaphragm are poorly visualized). The presence of hepatic steatosis in children with obesity was considered consistent with metabolic-associated steatotic liver disease.

Fasting blood samples were collected in the morning after an overnight fast to measure cholesterol, triglycerides, and liver enzymes, including alanine aminotransferase (ALT) and aspartate aminotransferase (AST), using standardized automated biochemical assays.

### 2.6. Outcome Measures

The primary outcome was biochemical response, defined for this retrospective analysis as a ≥30% reduction in serum ALT concentration from baseline to the 6–12-month follow-up assessment. This threshold was adopted as a categorical measure of substantial biochemical improvement, informed by previous pediatric MASLD studies demonstrating that meaningful improvement is accompanied by substantial reductions in ALT [58,59].

Secondary outcomes included changes in serum aspartate aminotransferase (AST), polysomnographic parameters (apnea–hypopnea index [AHI], REM AHI, NREM AHI, mean oxygen saturation, nadir oxygen saturation, and sleep efficiency), and hepatic steatosis grade on abdominal ultrasonography. Improvement in hepatic steatosis was defined as a decrease of at least one ultrasonographic grade from baseline to follow-up. Correlations between sleep-disordered breathing severity and liver injury markers were also evaluated.

### 2.7. Sample Size Calculation

As this was a retrospective cohort study using existing medical records, no a priori sample-size calculation was performed. All eligible children who received routine clinical care during the predefined study period and met the eligibility criteria were included. The final study population comprised 127 children, including 67 who had received targeted OSA treatment and 60 who had not received targeted OSA treatment. Complete baseline and 6–12-month follow-up data were available for all included participants.

### 2.8. Statistical Analysis

Statistical analyses were performed using IBM SPSS Statistics version 28 (IBM Corp., Armonk, NY, USA). Continuous variables were expressed as mean ± standard deviation (SD) or median (interquartile range), as appropriate, while categorical variables were presented as frequencies and percentages. Baseline characteristics were compared using the independent-samples *t*-test or Mann–Whitney *U* test for continuous variables and the χ^2^ test or Fisher’s exact test for categorical variables. Changes between baseline and follow-up within each treatment group were analyzed using paired *t*-tests or Wilcoxon signed-rank tests according to data distribution.

Direct between-group comparisons of continuous outcomes were performed using analysis of covariance (ANCOVA), with the follow-up value as the dependent variable and the corresponding baseline value and treatment group as covariates. Because treatment allocation was non-randomized and baseline OSA severity differed between groups, additional models incorporated clinically relevant baseline polysomnographic measures to evaluate the robustness of the observed associations. Treatment-by-baseline-characteristic interaction terms were examined when clinically appropriate. Adjusted effects are reported with 95% confidence intervals.

ALT change was also evaluated as a continuous outcome. Differences in ALT change among children receiving non-targeted treatment, adenotonsillectomy, CPAP, or combined adenotonsillectomy and CPAP were explored using one-way analysis of variance followed by post hoc pairwise comparisons. Because only three children received combined therapy, estimates for this subgroup were considered exploratory and interpreted cautiously.

Correlation analyses between polysomnographic parameters and liver enzymes were considered exploratory. Because multiple correlations were examined, individual *p* values were interpreted cautiously, and emphasis was placed on the magnitude and consistency of associations rather than isolated statistical significance. For the categorical biochemical-response analysis, multivariable binary logistic regression was used to explore factors associated with a ≥30% reduction in ALT from baseline to follow-up. Continuous predictor variables were dichotomized using prespecified clinically relevant thresholds to facilitate interpretation of odds ratios. Cutoff values were prespecified before analysis and selected according to internationally accepted pediatric OSA diagnostic criteria, established definitions of clinically significant nocturnal hypoxemia, and thresholds commonly used in previous pediatric polysomnographic studies [27,44,45]. A BMI z-score ≥ 2.5 was categorized using clinically relevant cutoffs that approximated the distribution of the study population and were selected to facilitate clinically interpretable regression analyses. A reduction in BMI z-score of ≥0.25 was included as a clinically relevant covariate in the multivariable analysis, based on previous evidence demonstrating that this degree of BMI z-score reduction is associated with greater improvements in serum aminotransferase levels in children [60].

Polysomnographic variables included the arousal index (≥10 events/h), limb movement index (≥5 events/h), apnea–hypopnea index (AHI ≥ 1 event/h), obstructive apnea index (≥1 event/h), hypopnea index (≥1 event/h), NREM AHI (≥1 event/h), REM AHI (≥1 event/h), mean nocturnal oxygen saturation (<92%), and nadir oxygen saturation (<90%). Polysomnographic parameters were assessed at baseline and at a single follow-up evaluation conducted 6–12 months later. Overnight polysomnography was repeated at follow-up using the same standardized scoring procedures as at baseline. Each participant therefore contributed one baseline and one follow-up PSG, and longitudinal changes in sleep architecture, respiratory indices, and nocturnal oxygenation were evaluated by comparing the follow-up values with the corresponding baseline measurements.

Treatment variables included adenotonsillectomy and continuous positive airway pressure (CPAP) therapy. Adjusted odds ratios (ORs) with 95% confidence intervals (CIs) were calculated. A two-sided *p* value < 0.05 was considered statistically significant.

## 3. Results

### 3.1. Study Population and Participant Flow

**Figure 1:**
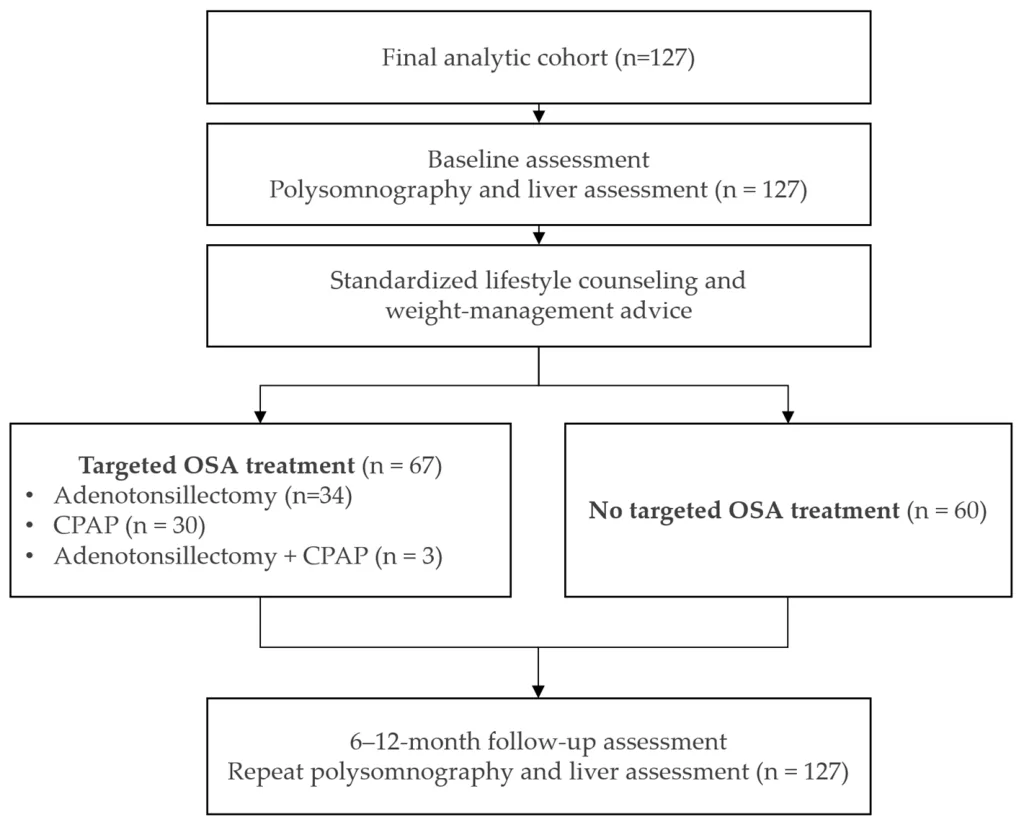
Study flow diagram showing participant selection, baseline and follow-up assessments, and classification according to OSA treatment status.

A total of 127 met the inclusion criteria and completed baseline and follow-up assessments (Figure 1). Of these, 67 (52.8%) received targeted OSA treatment, including adenotonsillectomy (*n* = 34), CPAP (*n* = 30), or both (*n* = 3), while 60 (47.2%) received standard care alone. Among children receiving CPAP therapy, the mean adherence rate was 84.7%, defined as CPAP use for at least 4 h per night on prescribed nights. The mean nightly duration of CPAP use was 7.7 h. The interval between baseline and follow-up polysomnography was comparable between groups, with a mean interval of 8.6 ± 1.6 months in the targeted-treatment group and 8.2 ± 1.2 months in the non-targeted-treatment group (mean difference, −0.40 months; 95% CI, −0.89 to 0.09; *p* = 0.11).

### 3.2. Baseline Clinical and Polysomnographic Characteristics

Baseline characteristics are summarized in Table 1. Demographic characteristics, anthropometric measurements, liver enzymes, lipid profiles, and hepatic steatosis grades were comparable between groups. The targeted treatment group had more severe sleep-disordered breathing, with significantly higher AHI and obstructive respiratory indices and lower nocturnal oxygen saturation than the non-treatment group (all *p* < 0.05). Sleep architecture was otherwise similar between groups.

### 3.3. Change in Clinical, Hepatic, and Polysomnographic Outcomes Following OSA Treatment

During the 6–12-month follow-up, the BMI z-score decreased modestly in both groups, with no significant between-group difference (targeted treatment: −0.16 vs. non-targeted treatment: −0.10; *p* = 0.13).

Targeted OSA treatment was associated with significant improvements in hepatic and sleep-related outcomes (Table 2). Children who received targeted therapy demonstrated significant reductions in both ALT and AST levels, along with improvement in ultrasonographic hepatic steatosis grading, whereas those without targeted treatment exhibited significant increases in liver enzyme levels and no improvement in hepatic steatosis.

Polysomnographic parameters also improved substantially following targeted treatment. Significant reductions were observed in AHI, obstructive apnea index, hypopnea index, NREM AHI, REM AHI, and arousal index, accompanied by improved nocturnal oxygenation (higher mean and nadir oxygen saturation; all *p* < 0.01). Sleep architecture also improved, with increased total sleep time and sleep efficiency and reduced NREM stage 1 sleep. In contrast, the non-targeted treatment group showed no significant improvement in OSA severity or oxygenation, while arousal index increased and nadir oxygen saturation declined during follow-up.

After adjustment for baseline ALT, the association between treatment and follow-up ALT remained statistically significant across models that were additionally adjusted for baseline AHI, average SpO_2_, or nadir SpO_2_ (all *p* < 0.01). Baseline AHI was not significantly associated with follow-up ALT (*p* = 0.12), whereas average SpO_2_ showed a modest significant association (*p* = 0.04); nadir SpO_2_ was not significantly associated with follow-up ALT (*p* = 0.49) (Table S1).

Between-group comparisons of the hepatic outcomes are presented in Table 3. Targeted OSA treatment was associated with significantly better ALT and ultrasonographic outcomes than non-targeted OSA treatment. ALT improvement was more frequent in the targeted group than in the non-targeted group (23.4% vs. 1.9%, *p* < 0.01), and follow-up ALT levels were significantly lower (31.8 vs. 44.6 U/L, *p* < 0.01), despite comparable baseline levels (*p* = 0.44). Similarly, ultrasonographic improvement was more frequent in the targeted group (22.0% vs. 10.2%, *p* = 0.02). Baseline hepatic steatosis grades were comparable between groups (*p* = 0.52), whereas follow-up grades were significantly lower in the targeted group (1.16 vs. 2.05, *p* < 0.01).

### 3.4. Correlation Between OSA Severity and Liver Injury

As shown in Table 4, indices of OSA severity were positively correlated with ALT and AST levels. AHI demonstrated the strongest association with liver injury, while lower nocturnal oxygen saturation was associated with higher liver enzyme concentrations. Sleep fragmentation, reflected by a higher arousal index, also showed a modest positive correlation with ALT.

### 3.5. Predictors of Improvement in Liver Injury

To evaluate improvement in liver injury, a reduction in serum ALT of ≥30% from baseline was predefined as the study outcome. During the follow-up period, 27 children achieved an ALT reduction of at least 30%, representing 40.3% of the treatment group. Among these participants, the mean change in ALT was −25.52 U/L, whereas children who did not achieve the predefined outcome demonstrated a mean increase of 3.22 U/L. The difference in ALT change between the two groups was statistically significant (*p* < 0.01).

In multivariable binary logistic regression analysis (Table 5a), CPAP therapy was associated with higher odds of achieving a ≥30% reduction in serum ALT during follow-up (OR 14.54, 95% CI 3.87–54.69; *p* < 0.01). The wide confidence interval indicates substantial imprecision in the effect estimate and warrants cautious interpretation. BMI z-score reduction ≥ 0.25 was not significantly associated with biochemical ALT response (OR 1.09, 95% CI 0.36–3.29; *p* = 0.88). No significant associations were observed for adenotonsillectomy, AHI, arousal index, limb movement index, or nadir oxygen saturation.

Subgroup analysis showed a significant difference in ALT change across the treatment groups (overall ANOVA, *p* < 0.01), with the greatest reduction observed in the CPAP group. Post hoc analysis showed greater ALT reduction with CPAP than with non-targeted treatment or adenotonsillectomy alone.

In an exploratory multivariable binary logistic regression analysis of ultrasonographic improvement (Table 5b), CPAP therapy was associated with higher odds of ultrasonographic improvement (OR 3.33, 95% CI 1.16–9.59; *p* = 0.03). Adenotonsillectomy (OR 1.02, 95% CI 0.40–2.62; *p* = 0.96), BMI z-score reduction ≥ 0.25 (OR 0.95, 95% CI 0.40–2.28; *p* = 0.91), limb movement index ≥ 5 events/h (OR 0.61, 95% CI 0.22–1.67; *p* = 0.34), and the other included polysomnographic variables were not significantly associated with ultrasonographic improvement.

## 4. Discussion

In this retrospective observational cohort study of children with obesity, OSA, and MASLD, receipt of targeted OSA treatment was associated with more favorable longitudinal changes in sleep-disordered breathing, nocturnal oxygenation, liver enzymes, and ultrasonographic hepatic steatosis over 6–12 months of follow-up. Children receiving adenotonsillectomy and/or CPAP demonstrated substantial reductions in OSA severity and improvements in nocturnal oxygenation, accompanied by decreases in ALT, AST, and hepatic steatosis grade, whereas children without targeted OSA treatment showed less favorable hepatic and polysomnographic trajectories. In exploratory treatment-modality analyses, the greatest reduction in ALT was observed among children receiving CPAP. These findings suggest an association between OSA-directed treatment and hepatic outcomes; however, because treatment was not randomly assigned, they should not be interpreted as evidence of a causal treatment effect.

The respiratory response observed after treatment is consistent with the established efficacy of OSA-directed therapy in children. Adenotonsillectomy has been shown to improve polysomnographic measures and symptoms in pediatric OSA, as demonstrated in multicenter and randomized studies, although residual disease is more frequent among children with obesity [49,50,51,52]. CPAP is an effective treatment for children with persistent or severe OSA when used consistently, and pediatric studies demonstrate improvement in respiratory physiology with adequate adherence [53,54]. Our findings extend this literature by demonstrating that longitudinal improvement in sleep-disordered breathing and nocturnal oxygenation was accompanied by favorable changes in biochemical and ultrasonographic hepatic outcomes.

The reduction in ALT and AST is clinically relevant because aminotransferases remain commonly used markers of hepatocellular injury in pediatric MASLD, despite imperfect sensitivity and specificity [7,12]. In the present study, children receiving targeted OSA treatment experienced improvement in liver enzymes, while untreated children showed persistent or worsening abnormalities. Because both groups received identical lifestyle counseling and weight-management advice, the divergence in hepatic outcomes suggests that alleviation of OSA-related physiological stress contributed to hepatic recovery. This interpretation is consistent with pediatric histologic studies showing links between OSA, hypoxemia, steatohepatitis, and fibrosis severity [44,45,46,55]. Nevertheless, differences in adherence to lifestyle recommendations and other unmeasured behavioral or metabolic factors could not be quantified retrospectively. Therefore, the observed differences should be interpreted as an association between receipt of OSA-directed treatment and more favorable biochemical liver outcomes rather than as evidence that OSA treatment directly produced hepatic recovery.

Both groups experienced modest reductions in BMI z-score during follow-up, and the magnitude of change did not differ significantly between the targeted-treatment and non-targeted-treatment groups. This observation suggests that differential weight change alone is unlikely to fully account for the more favorable hepatic outcomes observed in the targeted-treatment group. A BMI z-score reduction of ≥0.25 was included as a clinically relevant covariate because previous pediatric studies have associated this degree of weight reduction with greater improvements in aminotransferase levels [60]. However, BMI z-score reduction ≥ 0.25 was not independently associated with either biochemical ALT response (OR 1.09, 95% CI 0.36–3.29; *p* = 0.88) or ultrasonographic improvement (OR 0.95, 95% CI 0.40–2.28; *p* = 0.91) in the present study. Although these findings suggest that the observed hepatic associations were not explained by this degree of weight reduction, residual confounding from dietary adherence, physical activity, metabolic changes, and other unmeasured lifestyle factors cannot be excluded.

The longitudinal ultrasonographic findings provide an additional hepatic outcome beyond serum aminotransferase concentrations. Hepatic steatosis grade decreased during follow-up in children receiving targeted OSA treatment, whereas steatosis grade increased in those without targeted treatment. This directionally concordant change in biochemical and ultrasonographic outcomes strengthens the internal consistency of the observed association. However, conventional ultrasonography provides only a semiquantitative assessment of hepatic steatosis and cannot reliably quantify small changes in hepatic fat content or assess inflammation and fibrosis. Accordingly, improvement in ultrasonographic steatosis grade should not be interpreted as the histologic resolution of MASLD.

Several mechanisms may explain the hepatic benefit observed after OSA treatment. Intermittent hypoxia induces oxidative stress, mitochondrial dysfunction, inflammatory cytokine activation, and dysregulated lipid metabolism, all of which are central components of the multiple-hit model of MASLD pathogenesis [13,14,15,16,17,18,19,20,33,34,35]. Recurrent hypoxia–reoxygenation cycles activate hypoxia-inducible factor-1α (HIF-1α), nuclear factor-κB (NF-κB), and reactive oxygen species, promoting hepatocyte injury, insulin resistance, lipid peroxidation, and progressive hepatic steatosis [19,20,33,34,35,61,62]. OSA treatment reduces obstructive respiratory events and may reduce cumulative nocturnal hypoxic exposure, potentially attenuating these downstream inflammatory and metabolic pathways. The parallel improvements in nocturnal oxygenation and hepatic outcomes observed in this cohort are consistent with these proposed mechanisms, although the observational design does not establish a causal relationship between correction of sleep-disordered breathing and improvement in MASLD.

Exploratory correlation analyses provided additional evidence of relationships between sleep-disordered breathing and liver injury markers. Higher AHI and related respiratory indices were positively correlated with ALT and AST, whereas lower nocturnal oxygen saturation was associated with higher liver enzyme concentrations. Because multiple polysomnographic variables were examined and several were physiologically correlated, these analyses should be considered exploratory rather than confirmatory. Previous pediatric studies have demonstrated that nocturnal hypoxemia is particularly important in predicting steatohepatitis and fibrosis severity among children with fatty liver disease [44,45,46]. Similarly, adult studies and recent systematic reviews have shown that oxygen desaturation indices correlate more strongly with hepatic steatosis and fibrosis than AHI alone [21,22,23,24,36,37,38]. Together, these findings suggest that assessment of nocturnal oxygenation should complement conventional OSA severity indices when evaluating metabolic risk in children with obesity and MASLD.

In this present study, continuous polysomnographic variables were dichotomized using prespecified clinically relevant thresholds to facilitate interpretation of adjusted odds ratios and improve clinical applicability. These cutoffs were selected a priori based on established pediatric OSA diagnostic criteria, accepted definitions of nocturnal hypoxemia, and thresholds commonly used in previous pediatric sleep studies rather than being derived from the present dataset. Specifically, an AHI ≥ 1 event/h was used because it represents the diagnostic threshold for pediatric OSA according to American Academy of Sleep Medicine (AASM) scoring criteria and international pediatric OSA guidelines [27]. Likewise, mean nocturnal oxygen saturation <92% and nadir oxygen saturation <90% were chosen to identify clinically meaningful nocturnal hypoxemia, thresholds that have been widely used in pediatric sleep medicine and studies evaluating the cardiometabolic consequences of OSA [44,45].

Exploratory analyses according to OSA treatment modality demonstrated significant differences in ALT change, with the greatest reduction observed among children receiving CPAP. ALT decreased by approximately 17.8 U/L in the CPAP group compared with 5.6 U/L after adenotonsillectomy, whereas ALT increased by approximately 5.7 U/L among children receiving non-targeted treatment. Post hoc comparisons showed a greater ALT reduction with CPAP than with either non-targeted treatment or adenotonsillectomy alone. One possible explanation is that CPAP provides continuous upper-airway stabilization throughout sleep and may therefore provide more consistent control of residual obstructive events and nocturnal hypoxemia, whereas residual OSA after adenotonsillectomy is common among children with obesity [49,50,51,52]. In contrast, CPAP provides continuous stabilization of the upper airway throughout sleep, resulting in more consistent correction of intermittent hypoxemia and sleep fragmentation. Adult studies have similarly reported improvements in liver enzymes following effective CPAP therapy, although randomized trials have produced heterogeneous results owing to differences in treatment adherence, disease severity, and duration of therapy [39,40,41,42,43]. However, the wide confidence interval indicates substantial imprecision in the estimated magnitude of this association. Given the relatively small number of children receiving CPAP and the limited number of outcome events, model instability and overfitting cannot be excluded.

The favorable hepatic response observed in children receiving CPAP may partly reflect the excellent treatment adherence achieved in this cohort. Mean CPAP adherence reached 84.7% of nights with at least 4 h of use, while the average nightly usage was 7.7 h, substantially exceeding the conventional threshold for adequate adherence. Previous pediatric studies have consistently demonstrated that improvements in respiratory physiology, sleep quality, and cardiometabolic outcomes are highly dependent on CPAP adherence, with greater nightly use resulting in more effective correction of intermittent hypoxemia and sleep fragmentation [39,40,41,42,43,49]. The excellent adherence observed in our study likely contributed to the significant improvement in liver enzymes and supports the feasibility of long-term CPAP therapy within a multidisciplinary pediatric sleep program. However, the present observational data cannot determine whether greater CPAP exposure directly resulted in greater hepatic improvement. Future studies examining dose–response relationships between nightly CPAP use, reduction in hypoxic burden, and hepatic outcomes would help clarify this relationship.

The association between nocturnal oxygenation and ALT outcomes also warrants attention. In exploratory analyses, oxygenation measures were associated with biochemical liver outcomes, whereas AHI and several other conventional respiratory indices were not consistently associated after adjustment.

This finding supports the concept that correction of hypoxic burden, rather than reduction in respiratory event frequency alone, may be critical for improving biochemical markers of liver injury in children with obesity and MASLD. Although AHI remains the standard metric for diagnosing OSA, it does not fully capture the duration or severity of oxygen desaturation. Mean oxygen saturation integrates cumulative nocturnal hypoxic exposure, whereas AHI only counts respiratory events. Two children with identical AHI values may experience markedly different hypoxic burdens depending on event duration and desaturation depth. Therefore, hypoxic burden may more closely reflect the biological mechanisms driving oxidative stress, hepatocellular injury, and metabolic dysfunction. Recent evidence increasingly supports measures of hypoxic burden—including mean oxygen saturation, nadir oxygen saturation, and cumulative oxygen desaturation—as superior indicators of the cardiometabolic consequences of OSA [24,61,62,63,64]. Our findings extend this concept to pediatric MASLD and support the hypothesis that effective correction of nocturnal hypoxemia is a key mechanism underlying improvement in liver injury.

An important consideration is the substantial difference in baseline OSA severity between treatment groups. Children receiving targeted treatment had higher baseline AHI and lower nocturnal oxygen saturation, reflecting clinical selection of children with more severe disease for OSA-specific therapy. To address this imbalance, additional ANCOVA models were performed for follow-up ALT while accounting for baseline ALT and selected baseline polysomnographic measures. The association between treatment status and follow-up ALT remained evident in models incorporating baseline AHI and mean SpO_2_, whereas the association was attenuated when nadir SpO_2_ was considered. These findings suggest that the observed difference in ALT trajectory was not explained solely by baseline ALT; however, the variability across adjusted models also underscores the influence of baseline OSA severity and oxygenation. Because treatment allocation was non-randomized, these analyses reduce but cannot eliminate confounding by indication or regression to the mean.

The present study adds to the limited pediatric longitudinal literature. Most previous studies evaluating OSA and fatty liver disease in children were cross-sectional, limiting causal inference [44,45,46,47,48]. The current longitudinal design, objective polysomnography, and comparison of treated and untreated groups provide stronger evidence that modifying sleep-disordered breathing may influence hepatic outcomes. The findings are also aligned with the limited pediatric evidence suggesting improvement in NAFLD severity after treatment of OSA and nocturnal hypoxia [55].

From a clinical perspective, these findings have important implications for the multidisciplinary management of pediatric MASLD. Current clinical practice guidelines primarily emphasize lifestyle modification, dietary intervention, weight reduction, and the treatment of associated metabolic risk factors as the cornerstone of therapy [2,3,7,56,57]. However, sustained lifestyle modification remains challenging, and many children continue to demonstrate persistent liver enzyme abnormalities despite weight-management programs. Our results suggest that OSA represents an additional, potentially modifiable contributor to hepatic injury, particularly among children with obesity. Routine screening for sleep-disordered breathing should therefore be considered in children with obesity, persistent ALT elevation, or ultrasonographic evidence of MASLD, even in the absence of prominent daytime symptoms. Furthermore, objective assessment of nocturnal oxygenation may provide clinically meaningful information beyond conventional AHI measurements when identifying children at greatest risk of progressive liver disease. Integrating pediatric sleep medicine into multidisciplinary obesity and MASLD clinics may therefore improve risk stratification and expand therapeutic opportunities beyond lifestyle intervention alone.

The present study adds to the limited longitudinal pediatric literature examining OSA treatment and hepatic outcomes in children with obesity and MASLD. Its strengths include paired polysomnographic assessments, longitudinal biochemical and ultrasonographic liver evaluation, objective CPAP adherence monitoring, and comparison of children receiving and not receiving targeted OSA therapy. The ≥30% ALT reduction threshold was used as an operational categorical measure of substantial biochemical response rather than as a validated pediatric MASLD treatment endpoint; therefore, ALT was also evaluated as a continuous outcome.

The concordance of improvements in sleep-disordered breathing, nocturnal oxygenation, and liver enzymes, together with the independent associations of CPAP therapy and nocturnal oxygenation with ALT response, provides further evidence supporting OSA as a potentially modifiable contributor to pediatric MASLD. These findings underscore the importance of integrating pediatric sleep evaluation and treatment into multidisciplinary obesity and liver disease care.

Several limitations should be acknowledged. First, the observational design precludes definitive causal inference. Although logistic regression and ANCOVA sensitivity analyses were performed to account for measured baseline differences, residual confounding, particularly from unmeasured factors, cannot be excluded. Therefore, the findings should be interpreted as associations rather than definitive evidence of a causal treatment effect. Second, treatment allocation was based on routine clinical decision-making rather than randomization. Consequently, children receiving targeted OSA treatment had substantially more severe sleep-disordered breathing at baseline, introducing the potential for confounding by indication and regression-to-the-mean effects. Although multivariable analyses were performed to adjust for measured covariates, residual confounding cannot be excluded. Therefore, the observed associations should be interpreted as evidence of an association rather than definitive proof of causality. Third, hepatic outcomes were assessed using serum aminotransferases and ultrasonography rather than magnetic resonance imaging–proton density fat fraction (MRI-PDFF), transient elastography, or liver histology; consequently, changes in hepatic steatosis and fibrosis may have been underestimated. Fourth, although CPAP adherence was objectively monitored, the limited number of outcome events relative to the number of predictors included in the multivariable logistic regression models raises the possibility of model instability and overfitting. Accordingly, these analyses should be regarded as exploratory. In particular, the large odds ratio and wide confidence interval observed for CPAP in the ALT-response model indicate substantial imprecision and should be interpreted cautiously rather than as a precise estimate of treatment effect. Fifth, the duration of follow-up may not have been sufficient to determine whether improvements in biochemical markers translate into long-term reductions in hepatic fibrosis or other clinically important liver outcomes. Finally, this was a single-center and single-investigator study, which may limit the generalizability of the findings to other pediatric populations.

Despite these limitations, the study has several strengths. The longitudinal design allowed for paired assessment of sleep-disordered breathing and hepatic outcomes over a predefined 6–12-month follow-up interval. Objective polysomnography, standardized ultrasonographic assessment, complete follow-up data, and objective CPAP adherence monitoring strengthened the quality of the clinical data. The comparable follow-up interval between treatment groups further reduced the likelihood that differences in observation time accounted for the observed trajectories. In addition, evaluation of ALT as both a continuous outcome and a categorical biochemical response, together with adjusted analyses addressing baseline differences in OSA severity, provided complementary perspectives on the observed associations.

## 5. Conclusions

In this retrospective observational cohort of children with obesity, OSA, and MASLD, receipt of targeted OSA treatment was associated with more favorable changes in sleep-disordered breathing, nocturnal oxygenation, serum aminotransferase levels, and ultrasonographic hepatic steatosis over 6–12 months of follow-up. CPAP therapy was associated with a greater reduction in ALT, while a BMI z-score reduction of ≥0.25 was not independently associated with biochemical ALT response after multivariable adjustment. These findings suggest that improvement in OSA and nocturnal oxygenation may be associated with favorable hepatic outcomes beyond changes in weight alone. However, given the retrospective, non-randomized design and the potential for residual confounding, these associations should not be interpreted as evidence of a causal treatment effect. Prospective multicenter studies incorporating quantitative measures of hypoxic burden, advanced liver imaging, and longer follow-up are needed to determine whether treatment of OSA contributes to sustained improvement in pediatric MASLD.

## Figures and Tables

**Table 1:** Comparison of baseline characteristics between participants with and without targeted OSA treatment.

Variable	Targeted OSA Treatment (*n* = 67)	Non-Targeted OSA Treatment (*n* = 60)	*p* Value
Age (years)	8.9 ± 2.8	9.5 ± 2.7	0.12
Male sex, *n* (%)	52 (77.6)	43 (71.7)	0.44
Weight (kg)	60.9 ± 27.0	62.1 ± 22.6	0.41
Height (cm)	137.4 ± 17.9	142.6 ± 16.8	0.36
BMI (kg/m^2^)	30.8 ± 7.5	29.5 ± 5.1	0.05
BMI z-score	2.7 ± 0.5	2.5 ± 0.4	0.19
%BMI z-score	99.4 ± 0.6	99.1 ± 0.7	0.80
Diabetes Mellitus, *n* (%)	3 (4.5)	2 (3.3)	1.00
Hypertension, *n* (%)	4 (6.0)	10 (16.7)	0.05
Allergic Rhinitis, *n* (%)	49 (73.1)	42 (70.0)	0.70
Nasal Swelling			0.21
No swelling, *n* (%)	8 (11.9)	14 (23.3)	
Mild, *n* (%)	7 (10.4)	2 (3.3)	
Moderate, *n* (%)	28 (41.8)	26 (38.3)	
Severe, *n* (%)	24 (35.8)	20 (33.3)	
Very severe, *n* (%)	0 (0.0)	1 (1.7)	
Passive Smoking, *n* (%)	31 (46.3)	23 (38.3)	0.37
MAFLD by Liver Ultrasonogram			0.81
Grade 1, *n* (%)	30 (44.8)	30 (50.0)	
Grade 2, *n* (%)	26 (38.8)	22 (36.7)	
Grade 3, *n* (%)	11 (16.4)	8 (13.3)	
Alanine Aminotransferase (ALT, U/L)	42.4 ± 26.3	39.0 ± 22.5	0.09
Aspartate Aminotransferase (AST, U/L)	32.9 ± 15.7	32.5 ± 15.4	0.97
Total Cholesterol (mg/dL)	173.7 ± 33.0	166.2 ± 28.3	0.07
Triglyceride (mg/dL)	123.3 ± 53.5	125.1 ± 55.4	0.25
HDL Cholesterol (mg/dL)	48.2 ± 15.0	46.3 ± 10.9	0.66
LDL Cholesterol (mg/dL)	104.2 ± 27.7	97.5 ± 27.1	0.78
Adenoid-Nasopharyngeal Ratio (ANR)	0.7 ± 0.1	0.6 ± 0.2	0.05
Baseline Polysomnographic Parameters			
Total Sleep Time (TST, min)	433.1 ± 70.9	440.6 ± 60.5	0.60
Sleep Efficiency (%)	82.8 ± 10.3	84.2 ± 11.4	0.99
Sleep Latency (min)	18.8 ± 20.9	16.6 ± 15.0	0.06
REM Sleep Latency (min)	139.5 ± 79.5	173.0 ± 110.0	0.01
Sleep Stage NREM1 (%)	7.7 ± 5.4	6.5 ± 4.9	0.17
Sleep Stage NREM2 (%)	34.5 ± 12.4	38.4 ± 10.2	0.10
Sleep Stage NREM3 (%)	39.5 ± 12.7	38.5 ± 10.6	0.57
Sleep Stage REM (%)	18.0 ± 7.8	16.5 ± 6.5	0.11
Arousal Index	8.3 ± 11.4	5.9 ± 6.7	0.01
Limb Movement Index	5.6 ± 11.3	5.6 ± 14.1	0.80
Apnea–Hypopnea Index (AHI, events/h)	23.7 ± 32.0	9.3 ± 11.1	<0.01
Obstructive Apnea Index (OI, events/h)	11.5 ± 18.9	4.3 ± 6.6	<0.01
Hypopnea Index (HI, events/h)	11.5 ± 19.0	4.7 ± 7.5	<0.01
Central Apnea Index (CI, events/h)	0.4 ± 1.5	0.3 ± 0.9	0.38
NREM AHI (events/h)	24.5 ± 36.5	9.4 ± 14.5	<0.01
REM AHI (events/h)	26.4 ± 28.4	16.3 ± 20.8	0.01
Mean SpO_2_ (%)	93.1 ± 8.1	96.9 ± 1.6	<0.01
Nadir SpO_2_ (%)	67.8 ± 18.4	76.7 ± 14.4	0.04
Medical Treatment			
Intranasal Steroid, *n* (%)	56 (83.6)	44 (73.3)	0.16
Montelukast, *n* (%)	54 (80.6)	41 (68.3)	0.11
Antihistamine, *n* (%)	21 (31.3)	4 (6.7)	<0.01

Values are presented as mean ± standard deviation. BMI = body mass index; AHI = apnea–hypopnea index; SpO_2_ = nocturnal oxygen saturation; ALT = alanine aminotransferase; AST = aspartate aminotransferase.

**Table 2:** Parameter changes after 6–12 months (BMI z-score, liver enzymes, and polysomnographic data).

Variable	Baseline	Follow-Up	Mean Change	*p* Value
**Targeted OSA Treatment**				
BMI Z-Score	2.7 ± 0.5	2.6 ± 0.5	−0.16	<0.01
ALT (U/L)	42.4 ± 26.3	31.8 ± 17.7	−10.6	<0.01
AST (U/L)	32.9 ± 15.7	27.9 ± 11.6	−4.9	<0.01
Hepatic steatosis grade 1–3	1.7 ± 0.7	1.2 ± 0.7	−0.6	<0.01
Total Sleep Time (TST, min)	432.9 ± 71.4	464.1 ± 38.5	31.2	<0.01
Sleep Efficiency (%)	82.9 ± 10.4	87.0 ± 8.2	4.1	0.02
Sleep Latency (min)	18.8 ± 20.9	24.2 ± 17.1	5.4	0.10
REM Sleep Latency (min)	137.5 ± 78.5	152.2 ± 62.3	14.7	0.22
Sleep Stage NREM1 (%)	7.5 ± 5.3	5.4 ± 3.2	−2.1	0.01
Sleep Stage NREM2 (%)	34.5 ± 12.5	38.7 ± 10.4	4.2	0.02
Sleep Stage NREM3 (%)	39.8 ± 12.6	36.3 ± 11.8	−3.5	0.08
Sleep Stage REM (%)	18.2 ± 7.7	19.3 ± 4.8	1.1	0.34
Arousal Index	8.4 ± 11.5	6.1 ± 5.4	−2.3	0.06
Limb Movement Index	5.7 ± 11.4	3.5 ± 4.4	−2.1	0.13
Apnea–Hypopnea Index (AHI, events/h)	24.0 ± 32.2	3.2 ± 7.4	−20.7	<0.01
Obstructive Apnea Index (OI, events/h)	11.6 ± 19.0	1.6 ± 3.6	−10.0	<0.01
Hypopnea Index (HI, events/h)	11.6 ± 19.1	1.6 ± 4.1	−10.1	<0.01
Central Apnea Index (CI, events/h)	0.4 ± 1.5	0.1 ± 0.2	−0.3	0.07
NREM AHI (events/h)	24.7 ± 36.7	3.1 ± 8.6	−21.6	<0.01
REM AHI (events/h)	26.7 ± 28.5	5.7 ± 12.0	−21.0	<0.01
Mean SpO_2_ (%)	93.2 ± 8.1	97.8 ± 1.0	4.6	<0.01
Nadir SpO_2_ (%)	67.8 ± 18.4	84.5 ± 5.5	16.7	<0.01
**Non-Targeted OSA Treatment**				
BMI Z-Score	2.5 ± 0.4	2.4 ± 0.4	−0.10	<0.01
ALT (U/L)	38.9 ± 22.5	44.6 ± 21.1	5.65	<0.01
AST (U/L)	32.5 ± 14.4	36.4 ± 14.6	3.9	0.02
Hepatic steatosis grade 1–3	1.6 ± 0.7	2.1 ± 0.7	0.4	<0.01
Total Sleep Time (TST, min)	440.6 ± 60.5	447.4 ± 50.8	6.8	0.46
Sleep Efficiency (min)	84.2 ± 11.4	86.3 ± 7.6	2.1	0.24
Sleep Latency (min)	16.6 ± 15.0	21.9 ± 15.2	5.4	0.07
REM Sleep Latency (min)	173.0 ± 110.0	141.1 ± 66.4	−31.9	0.07
Sleep Stage NREM1 (%)	6.5 ± 4.9	6.3 ± 3.2	−0.3	0.74
Sleep Stage NREM2 (%)	38.4 ± 10.2	33.3 ± 10.9	−5.1	0.01
Sleep Stage NREM3 (%)	38.5 ± 10.6	42.7 ± 11.7	4.2	0.05
Sleep Stage REM (%)	16.5 ± 6.5	17.9 ± 5.8	1.4	0.12
Arousal Index	5.9 ± 6.7	10.5 ± 8.4	4.6	<0.01
Limb Movement Index	5.6 ± 14.1	6.1 ± 6.9	0.5	0.78
Apnea–Hypopnea Index (AHI, events/h)	9.3 ± 11.1	10.8 ± 8.3	1.5	0.16
Obstructive Apnea Index (OI, events/h)	4.3 ± 6.6	4.6 ± 5.5	0.3	0.77
Hypopnea Index (HI, events/h)	4.7 ± 7.5	6.3 ± 6.9	1.6	0.07
Central Apnea Index (CI, events/h)	0.3 ± 0.9	0.1 ± 0.1	−0.2	0.09
NREM AHI (events/h)	9.4 ± 14.5	10.4 ± 8.7	1.0	0.52
REM AHI (events/h)	16.3 ± 20.8	19.5 ± 16.8	3.2	0.15
Mean SpO_2_ (%)	96.9 ± 1.6	96.3 ± 2.3	−0.5	0.15
Nadir SpO_2_ (%)	76.7 ± 14.4	70.2 ± 13.0	−6.5	0.01

Data are presented as mean ± SD. *p* values were calculated using paired *t*-tests.

**Table 3:** Comparison of ALT and ultrasonographic outcomes between targeted and non-targeted OSA treatment groups.

Parameter	Targeted OSA Treatment	Non-Targeted OSA Treatment	*p* Value	95% Confidence Interval
**ALT**			<0.01	
ALT improved (*n*, %)	25 (23.4%)	2 (1.9%)		
ALT not improved (*n*, %)	42 (39.3%)	58 (54.2%)		
Mean ALT (U/L), baseline	42.4	39.0	0.44	−12.07–5.23
Mean ALT (U/L), follow-up	31.8	44.6	0.00	5.99–19.63
**Ultrasonographic Improvement**			0.02	
Ultrasonographic improved (*n*, %)	28 (22.0%)	13 (10.2%)		
Ultrasonographic not improved (*n*, %)	39 (30.7%)	47 (37.0%)		
Mean hepatic steatosis grade 1–3, baseline	1.72	1.63	0.52	−0.34–0.17
Mean hepatic steatosis grade 1–3, follow-up	1.16	2.05	<0.01	0.63–1.14

**Table 4:** Correlation between sleep severity and liver injury markers.

Variable	ALT r	*p* Value	AST r	*p* Value
Total Sleep Time (TST, min)	−0.08	0.39	−0.14	0.12
Sleep Efficiency (%)	0.01	0.89	0.02	0.82
Sleep Latency (min)	−0.09	0.32	−0.13	0.16
REM Sleep Latency (min)	−0.01	0.95	0.04	0.64
Sleep Stage NREM1 (%)	−0.08	0.37	0.05	0.60
Sleep Stage NREM2 (%)	−0.10	0.25	−0.14	0.12
Sleep Stage NREM3 (%)	0.18	0.04	0.14	0.10
Sleep Stage REM (%)	−0.19	0.03	−0.08	0.38
Arousal Index	0.22	0.01	0.10	0.24
Limb Movement Index	0.14	0.12	0.04	0.65
Apnea–Hypopnea Index (AHI, events/h)	0.32	<0.01	0.30	<0.01
Obstructive Apnea Index (OI, events/h)	0.21	0.02	0.30	<0.01
Hypopnea Index (HI, events/h)	0.30	0 < 0.01	0.21	0.02
Central Apnea Index (CI, events/h)	−0.06	0.49	−0.08	0.38
NREM AHI (events/h)	0.30	<0.01	0.26	<0.01
REM AHI (events/h)	0.31	<0.01	0.28	<0.01
Mean SpO_2_ (%)	−0.06	0.49	−0.18	0.04
Nadir SpO_2_ (%)	−0.19	0.03	−0.25	0.01

Pearson correlation coefficients are presented.

**Table 5:** (**a**) Multivariate binary logistic regression analysis of factors associated with biochemical ALT response (≥30% reduction). (**b**) Multivariable binary logistic regression analysis of factors associated with ultrasonographic improvement in liver steatosis.

(a)			
Variable	Odds Ratio (OR)	95% Confidence Interval	*p* Value
Arousal Index (≥10 events/h)	0.35	0.10–1.31	0.12
Limb Movement Index (≥5 events/h)	1.54	0.45–5.29	0.50
Apnea–Hypopnea Index (AHI, ≥1 event/h)	2.04	0.60–6.91	0.25
Nadir SpO_2_ (<90%)	3.42	0.98–11.97	0.05
BMI Z-Score Reduction (≥0.25)	1.09	0.36–3.29	0.88
Adenotonsillectomy	2.73	0.79–9.41	0.11
CPAP	14.54	3.87–54.69	<0.01
**(b)**			
**Variable**	**Odds Ratio (OR)**	**95% Confidence Interval**	***p*** ** Value**
Arousal Index (≥10 events/h)	1.76	0.62–5.00	0.29
Limb Movement Index (≥5 events/h)	0.61	0.22–1.67	0.34
Apnea–Hypopnea Index (AHI, ≥1 event/h)	1.18	0.42–3.29	0.75
Nadir SpO_2_ (<90%),	2.58	0.89–7.50	0.08
BMI Z-Score Reduction (≥0.25)	0.95	0.40–2.28	0.91
Adenotonsillectomy	1.02	0.40–2.62	0.96
CPAP	3.33	1.16–9.59	0.03

Note: Achievement of a biochemical ALT response, defined as a ≥30% reduction in serum ALT concentration from baseline to follow-up. Continuous variables were dichotomized using prespecified clinically relevant thresholds based on pediatric OSA diagnostic criteria and accepted clinical oxygenation thresholds. Ultrasonographic improvement was defined as a better liver ultrasound grade at follow-up and coded as 1 = improved and 0 = not improved or worse. BMI z-score reduction > 0.25 was coded as 1 = reduction and 0 = reduction ≤ 0.25 or increase. OR > 1 indicates higher odds of ultrasonographic improvement.

## Data Availability

The data presented in this study are available upon request from the corresponding author. The data are not publicly available due to privacy and ethical reasons.

## Supplementary Materials

The following supporting information can be downloaded at https://www.mdpi.com/article/10.3390/children13091241/s1, Table S1. Analysis of Covariance (ANCOVA) of Follow-up ALT Adjusted for Baseline OSA Severity and Nocturnal Oxygenation.

## Abbreviations

AHI	Apnea–Hypopnea Index
ALT	Alanine Aminotransferase
AST	Aspartate Aminotransferase
BMI	Body Mass Index
CI	Confidence Interval
CPAP	Continuous Positive Airway Pressure
IRB	Institutional Review Board
MASLD	Metabolic Dysfunction-Associated Steatotic Liver Disease
NREM	Non-Rapid Eye Movement
OAI	Obstructive Apnea Index
OR	Odds Ratio
OSA	Obstructive Sleep Apnea
PSG	Polysomnography
REM	Rapid Eye Movement
SpO_2_	Peripheral Oxygen Saturation
TCTR	Thai Clinical Trials Registry
TST	Total Sleep Time
WHO	World Health Organization
