# Supplementary material for: Outcome of Targeted Therapy for Obstructive Sleep Apnea in Children with Obesity and Metabolic Dysfunction-Associated Steatotic Liver Disease (MASLD)
Source: Children (Basel). 2026 Sep 14;13(9):1241. doi: 10.3390/children13091241 (PMC13605578; doi:10.3390/children13091241)
Supplement: Supplementary file 1 [file children-13-01241-s001.zip › children-4515052-supplementary.pdf]

Table S1. Analysis of Covariance (ANCOVA) of Follow-up ALT Adjusted for Baseline OSA Severity and Nocturnal Oxygenation.

Model	Covariate	F	*p* Value	Treatment F	Adjusted Difference, Treated vs. Untreated
1	Baseline AHI	2.44	0.121	56.93	−14.28 (−18.03 to −10.54)
2	Baseline Mean SpO_2	4.31	0.040	54.44	−13.94 (−17.68 to −10.20)
3	Nadir SpO_2	0.48	0.488	60.84	−14.77 (−18.52 to −11.02)
